# Author Correction: Development of a multivariable prediction model for severe COVID-19 disease: a population-based study from Hong Kong

**DOI:** 10.1038/s41746-022-00586-w

**Published:** 2022-03-28

**Authors:** Jiandong Zhou, Sharen Lee, Xiansong Wang, Yi Li, William Ka Kei Wu, Tong Liu, Zhidong Cao, Daniel Dajun Zeng, Keith Sai Kit Leung, Abraham Ka Chung Wai, Ian Chi Kei Wong, Bernard Man Yung Cheung, Qingpeng Zhang, Gary Tse

**Affiliations:** 1 School of Data Science, City University of Hong Kong, Hong Kong, China; 2 Cardiovascular Analytics Group, Laboratory of Cardiovascular Physiology, Hong Kong, China; 3 Li Ka Shing Institute of Health Sciences, Hong Kong, China; 4 Department of Cardiothoracic Surgery, Wuhan Asia Heart Hospital Affiliated to Wuhan University of Science and Technology, Hubei, Wuhan, China; 5 Tianjin Key Laboratory of Ionic-Molecular Function of Cardiovascular Disease, Department of Cardiology, Tianjin Institute of Cardiology, Second Hospital of Tianjin Medical University, Tianjin, China; 6 Institute of Automation, Chinese Academy of Sciences, Beijing, China; 7 Emergency Medicine Unit, LKS Faculty of Medicine, University of Hong Kong, Pokfulam, Hong Kong, China; 8 Department of Pharmacology and Pharmacy, University of Hong Kong, Pokfulam, Hong Kong, China; 9 Medicines Optimisation Research and Education (CMORE), UCL School of Pharmacy, London, United Kingdom; 10 Department of Medicine, University of Hong Kong, Pokfulam, Hong Kong, China

**Keywords:** Risk factors, Medical research

Correction to: *npj Digital Medicine* 10.1038/s41746-021-00433-4, published online 08 April 2021

In this article, the affiliation details for Gary Tse were incorrectly given as ‘Department of Cardiothoracic Surgery, Wuhan Asia Heart Hospital Affiliated to Wuhan University of Science and Technology, Hubei, Wuhan, China’, but should have been ‘Tianjin Key Laboratory of Ionic-Molecular Function of Cardiovascular Disease, Department of Cardiology, Tianjin Institute of Cardiology, Second Hospital of Tianjin Medical University, Tianjin 300211, China’. The original article has been corrected.”

